# Disaggregation of between- and within-subject effects of internalizing symptoms on P300 amplitude during adolescence

**DOI:** 10.1017/S0954579426101369

**Published:** 2026-04-28

**Authors:** Nicholas J. Santopetro, C.J. Brush, Alexander Kallen, Nader Amir, Greg Hajcak

**Affiliations:** 1 Psychology, Florida State University, Tallahassee, USA; 2 School of Kinesiology, Auburn University, USA; 3 Psychology, San Diego State University, USA; 4 School of Education and Counseling Psychology, Santa Clara University, USA

**Keywords:** adolescence, anxiety, depression, EEG, ERP, P300

## Abstract

Deviations in P300 activity have been implicated in depression and anxiety; however, much of this research has been conducted in adult samples and has primarily examined the association between P300 amplitude and internalizing symptoms *between participants*. We sought to simultaneously examine the between- and within-subject associations between depression and anxiety symptoms with P300. Self-report and neural data from a flanker task were collected at three timepoints over the course of two years in a large sample of adolescents (*n* = 490). *Blunted* P300 was robustly related to elevated between-subject depression. Conversely, elevations in within-subject anxiety were associated with *larger* P300. Results implicate the P300 as a reliable correlate of between-subjects level depression-related deficits in cognitive functions that is not susceptible to within-subject changes. Additionally, P300 also serves as a correlate of within-subject elevations in youth anxiety symptoms likely reflecting greater hyperarousal at the time of assessment.

## Introduction

Depressive and anxiety disorders are prevalent and debilitating mental health issues afflicting adolescents. Recent research has observed that rates of these disorders have been further impacted by the COVID-19 pandemic, with an estimated 21% to 25% of youth suffer from these significant internalizing problems (Racine et al., 2021). There are significant burdens linked with these internalizing disorders as adolescents experiencing depression are at an increased risk of experiencing suicidal ideations and behaviors, engaging in harmful substance use, and are more likely to exhibit social and educational impairments (Petito et al., 2020). Adolescents suffering from anxiety disorders are more likely to experience inattentiveness and concentration difficulties, academic problems, poor peer relations, low self-esteem, and low social competence (Farrell & Barrett, 2007). Additionally, adolescents experiencing comorbid depression and anxiety experience poorer treatment outcomes (Cummings et al., 2014; Schleider et al., 2014; Young et al., 2006; O’Neil & Kendall, 2012).

Adding to this burden, both internalizing disorders are also highly comorbid making it difficult to determine which disorder should be the primary target for interventions in mental health treatment settings. Indeed, Clark and Watson (1991) posited that a tripartite model exists between depression and anxiety such that both disorders are characterized by core symptoms of negative affectivity but diverge from one another in terms of symptoms related to dysfunctions in positive affectivity, more unique to depression, and excessive hyperarousal which is more unique to anxiety. It is therefore imperative to uncover reliable markers unique to depressive and anxiety disorders that could be leveraged to parse these disorders more effectively to better inform treatment strategies as well as increase our overall understanding of the pathoetiologies of these separate mental health conditions. Currently, the *distinct* neurological underpinnings which differentiate depressive and anxiety disorders are not well understood especially during adolescence.

ERP research can assist in the identification of these neural characteristics that uniquely define depression apart from anxiety, or vice versa (Hajcak et al., 2019). Briefly, ERPs are direct measures of brain activity recorded at the scalp via EEG during psychological tasks. One particular ERP component that can be employed to assist in unraveling the overlap that exists between these internalizing disorders is the P300 component. The P300 is a positive going component that is typically maximal over centroparietal regions of the scalp and occurs roughly 300 ms after stimulus presentation in an experimental task (e.g., oddball, go/no-go, or flanker). The functionality of the P300 is theorized to reflect general cognitive processes such as attentional allocation, context updating, and inhibitory control (Polich, 2012; Hajcak & Foti, 2020). In line with this proposed functionality, past research has demonstrated that the P300 is sensitive to task demands and motivational salience of stimuli employed in paradigms. Stimuli salience can be modulated by factors such as the context of the task. For example, participants are asked to respond to an infrequent target stimulus and ignore a more frequent standard stimulus in the classic oddball paradigm resulting in larger P300 to target stimuli as they are more demanding (i.e., require a swift response) while a smaller P300 to standards is observed as the stimuli is less salient and require less cognitive resources (Polich & Margala, 1997). Therefore, the P300 can serve as a suitable candidate to better understand underlying cognitive and motivational deviations associated with depression and anxiety symptomology.

In adult samples, P300 amplitude measured during visual or auditory oddball tasks is commonly found to be significantly reduced in those with depression (Bruder et al., 2009; Gangadhar et al., 1993; Nan et al., 2018; Roschke & Wagner, 2003; Urretavizcaya et al., 2003; Zhou et al., 2019). These findings have recently been replicated in studies eliciting P300 from the fast-paced flanker task (Eriksen & Eriksen, 1974), in which clinically depressed adults were characterized by smaller P300 amplitude compared to controls with no history of psychopathology (Klawohn, Santopetro et al., 2020). Additionally, the P300 elicited from flanker stimuli was associated with increases in depression symptoms over nine months in adults suffering from depressive disorders (Santopetro et al., 2021). Similar observations exist in the smaller P300 and adolescent depression literature. Past researchers have observed reductions in P300 associated with history of depression in a large sample of female adolescents that completed a complex visual oddball task (Houston et al., 2003). More recent investigations observed smaller P300 amplitude, elicited from the flanker task, predicted increases in depression severity over two years in a sample of female adolescents (Santopetro et al., 2020), and that reduced P300 related to elevations in current depressive symptoms (Santopetro et al., 2022; Thompson et al., 2023). In sum, the majority of this past research suggests that deficits in P300 amplitude, indexing impairments in processing of imperative target stimuli, are likely reflecting broad cognitive and motivational dysfunctions that are common in depressive disorders (Bruder et al., 2012; American Psychiatric Association, 2013; Santopetro et al., 2025).

Findings regarding P300 amplitude and anxiety disorders in adult samples are less consistent compared to the adult depression literature. Some researchers find that P300 amplitude is *larger* in individuals with a lifetime diagnosis of an anxiety disorder compared to individuals with no history of anxiety disorders (Enoch et al., 2001), and that individuals suffering *primarily* from an anxiety disorder (i.e., “pure” anxiety) are characterized by larger P300 compared to other clinical groups and a control group (Bruder et al., 2002; Enoch et al., 2008). These researchers posited that enhanced P300 amplitude in anxiety might reflect a trait-like marker of excessive attention allocation. Conversely, there is more recent research that does not observe significant P300 amplitude differences in individuals suffering from GAD compared to controls (Yang et al., 2015), or between individuals with GAD compared to individuals suffering from comorbid GAD and depression (Zhou et al., 2023). Further, there are also studies suggesting that individuals with diagnoses of anxiety disorders exhibit *smaller* P300 amplitude compared to controls (Bauer et al., 2001).

Comparable to the adolescent depression and P300 literature, less research has investigated the relationship between P300 amplitude and anxiety in adolescents. The studies that do investigate this relationship seem to demonstrate conflicting findings similar to the adult anxiety and P300 literature; some researchers observe a *blunted* P300 amplitude related to anxiety in youth (Éismont et al., 2009), others report *larger* P300 amplitude associated to youth anxiety symptoms (Singhal et al., 2012), and there is evidence positing no significant P300 amplitude differences in children or adolescents with anxiety (Hogan et al., 2007). Adding to these findings, recent work suggested that adolescents with heightened symptoms of anxiety exhibited reductions in flanker P300 amplitude; however, this initial association was better explained by elevated symptoms of depression which were overlapping with anxiety symptoms (Santopetro et al., 2021). Together, these findings regarding anxiety and P300 amplitude in both adult and adolescent samples are mixed, leaving our comprehension of the functionality of the P300 in these disorders inconclusive. Simultaneously examining the association of P300 with these often co-occurring internalizing symptoms is rarely done and findings from such investigations could assist in clarifying the distinct relationships between P300 with depression and anxiety.

The bodies of literature reviewed above overwhelming consist of studies that examined *between-subject level* associations (i.e., comparing an individual’s mean symptoms to another individual’s mean symptoms) of internalizing symptoms with P300 amplitude utilizing cross-sectional analyses *consisting of just one time point* leaving our understanding of how within-subject fluctuations (i.e., symptom changes relative to the individual’s *personal* symptom mean) of these internalizing symptoms impact the P300 largely unknown. Longitudinal data collected from the same individuals across multiple timepoints in combination with advanced statistical methods are necessary to examine within-subject relationships between neural activity and internalizing symptoms more precisely. Specifically, multilevel modeling (MLM) is a statistical approach that can be used to account for nested study designs (e.g., longitudinal studies) and separate between- and within-subject sources of variability in relationships between internalizing symptoms and P300 amplitude (e.g., Curran & Bauer, 2011; Wang & Maxwell, 2015). To our knowledge, using MLM to disaggregate both between- and within-subject relationships between internalizing symptoms and P300 amplitude has never been tested. Results from such models would shed further light on whether deviations in P300 amplitude are more sensitive to the effects of depression or anxiety, and if these unique associations are broadly stable longitudinally (i.e., between-subject effect), or whether they vary by occasion (i.e., within-subject effect). Such findings could clarify discrepant results in the internalizing and P300 literature as well as further cement our conceptualization of state- and trait-like deviations in cognitive processes that uniquely contribute to depression and anxiety. Moreover, understanding these distinctions (i.e., both the discrete influences of depression and anxiety as well as between- and within-subjects effects) can better inform case conceptualizations leading to more targeted and effective treatment plans.

Therefore, the present study sought to simultaneously examine the between- and within-subject relationships between adolescent depressive and anxiety symptoms with flanker P300 amplitude collected during a large longitudinal study. More specifically, male and female adolescents participated in a two-year study consisting of three visits: a baseline visit, a two-month follow-up visit, and a two-year follow-up visit. At each visit, the P300 was collected from the flanker task while continuous EEG was recorded. Participants also completed self-report questions regarding current depressive and anxiety symptoms. We hypothesize that reductions in P300 amplitude will be uniquely associated to between-subject depressive symptomology, not anxiety symptoms. We do not have any specific hypotheses regarding the associations between within-subject variance in internalizing symptoms with P300 amplitude during adolescence.

## Method

### Participants

The present study was part of a large multi-site longitudinal project examining the effectiveness of a computerized adaptive attention bias modification (AABM) training at modifying symptoms of anxiety as well as neural activity associated with errors (i.e., the error-related negativity [ERN]) in adolescents. The findings regarding the primary aims of this project (Amir et al., 2024), and a recent study focused on the cross-sectional relationship between stimulus-locked neural activity and internalizing symptoms collected at the baseline visit (Santopetro et al., 2022) are reported elsewhere. A total of 645 adolescent participants aged 11 to 14 years and their caregivers were recruited to participate in the baseline visit (T1) and were then invited back to complete both a two-month (T2) and two-year (T3) follow-up visit. Participants and their caregivers were initially recruited via word-of-mouth, registration lists, and online advertisements (e.g., Facebook). Participants completed a battery of computerized self-report questionnaires, clinical interviews, and various computerized tasks while continuous EEG recorded at each of the assessments. The inclusion criteria for this project included fluency in English and the presence of a parent or legal guardian at the lab visit, while exclusion criteria included the presence of a significant developmental or medical disability, a history of severe head injury, color blindness, or severe risk for suicide. Participating families provided both informed consent and assent prior to participating in any study protocols and were compensated for their time at each assessment. The study was approved by the Institutional Review Boards at Stony Brook University, Florida State University, and San Diego State University.

The present study utilized adolescent self-report data and stimulus-locked (i.e., flanker) EEG data collected during a computerized flanker task collected at the baseline (T1), two-month (T2) and two-year (T3) follow-up visits. Of the initial 645 participants that completed the baseline visit (T1), 13 participants were excluded for poor EEG data based on visual inspection and 9 were excluded due to poor performance on the flanker task (i.e., error rates more than 3 standard deviations from the mean). An additional four participants were missing self-report data. The average age of the sample at baseline was 12.93 years (*SD* = 1.14), with a fairly even sex split (i.e., 48% female). A majority of participants identified as White (59%), with the remainder identifying as Hispanic (22%), African American (9%), Asian (3%), or other (7%). A total of 23 participants did not answer their ethnicity or race at baseline. Average household income of families recruited from the Long Island, NY and Tallahassee, FL area at baseline was $105,323 (*SD* = 114,374); household income was not collected in the San Diego sample.

A total of 471 families returned for the two-month follow-up visit (T2). Of these 471 participants, 462 participants completed the flanker task during continuous EEG recording. From these 462 participants, 10 participants were excluded for poor EEG data based on visual inspection, and 3 were excluded due to poor performance on the flanker task (i.e., error rates more than 3 standard deviations from the mean). Additionally, 14 participants were missing self-report data on either current depressive or anxiety symptoms at this follow-up visit. The average age of our sample at the two-month follow-up visit was 13.12 years (*SD* = 1.14), with a fairly even sex split (i.e., 47% female).

Lastly, 488 participants returned for the two-year follow-up visit (T3); however, due to the COVID-19 pandemic, only 204 participants of these participants were able to complete the in-person study visit in which they were able to complete the flanker task while EEG recorded. The remaining 284 participants completed a virtual two-year follow-up visit consisting of online self-report questionnaires; these participants did not complete the flanker task. Of the 204 participants that completed the in-person two-year follow-up visit before the COVID-19 pandemic-related shut down, 4 participants were excluded for poor EEG data based on visual inspection, and one was excluded due to poor performance on the flanker task (i.e., error rate more than 3 standard deviations from the mean). Thus, the final sample for two-year follow-up analyses included 483 participants (199 participants with EEG data). The average age of our sample at the two-year follow-up visit was 14.93 years (*SD* = 1.17), with a fairly even sex split (i.e., 46% female).

For the purposes of this study, participants were included in the final analyses if they had usable EEG data from at least two of the three timepoints as MLM is robust and can handle missing values in outcome variables. Therefore, the total sample comprised of 490 adolescents. Considering that 62% of this sample completed the AABM training over the course of 8 weeks (i.e., the time period between T1 to T2), AABM training status (0 = no training received, 1 = training received) will be included as an additional fixed-effect variable in sensitivity analyses. Table 1 has more detailed information regarding our final sample at each assessment.

**Table 1.** Demographic, clinical, and P300 measures of the present sample at the baseline (T1), two-month (T2), and two-year (T3) visits

	Baseline (T1)	Two-month (T2)	Two-year (T3)
*Demographics and clinical*			
Age (Years)	12.95 (1.15)	13.13 (1.14)	14.98 (1.16)
Sex (% female)	46%	47%	45%
Race (%)			
White	56%	57%	53%
Hispanic	23%	22%	26%
Black/African American	7%	8%	5%
Asian	4%	3%	4%
Other	8%	8%	7%
CDI total score	7.42 (6.96)	5.65 (6.39)	8.19 (7.51)
SCARED total score	20.33 (13.30)	16.51 (12.85)	19.77 (14.18)
*ERP*			
P300 (μV)	14.60 (6.76)	10.84 (6.25)	10.32 (5.27)

*Note.* Means are presented, standard deviations in parentheses. CDI represents the Child’s Depression Inventory. SCARED represents the Screen for Child Anxiety Related Emotional Disorders.

### Measures

#### Children’s depression inventory (CDI)

The CDI is comprised of a total of 27 items that assess depressive severity in children and adolescents over the most recent two-week period (Kovacs, 1992). These items are scored on a 3-point Likert scale ranging from no presence of the symptom (0), mild presentation of the symptom (1), and severe symptoms (2). Past psychometric research suggests the CDI exhibits adequate internal consistency and convergent validity with clinical depressive disorders in various adolescent samples (Ivarsson et al., 2006; Rivera et al., 2005). In the present sample, the CDI total score exhibited excellent internal consistency at each assessment according to Cronbach’s alpha values (*T1*: .90; *T2*: .91; *T3*: .91). Lastly, CDI total score demonstrated good test-retest reliability both over a two-month period (*r* = .79; T1 to T2) and a two-year period (*r* = .61; T1 to T3).

#### Screen for child anxietyrelated emotional disorders (SCARED)

The SCARED is an anxiety measure that is comprised of 41 items that assesses various anxiety symptoms in children and adolescents (Birmaher et al., 1997). These items are scored on a 3-point Likert scale as either 0 (“not true or hardly ever true”), 1 (“somewhat true or sometimes true”), or 2 (“very true or often true”). Past research has demonstrated that the SCARED exhibits good internal consistency (Angulo et al., 2017; Birmaher et al., 1999). In the current sample, Cronbach’s alpha for the SCARED total score was excellent at each assessment (*T1*: .93; *T2*: .94; *T3*: .94). The SCARED total score also exhibited good test-retest reliability both over a two-month period (*r* = .77; T1 to T2) and a two-year period (*r* = .63; T1 to T3).

### Procedures

#### Flanker task

Participants completed a computerized arrowhead version of the flanker task at each assessment. Participants were presented with five horizontal arrow heads for 200 ms on each trial and the inter-trial interval (ITI) varied from 2,300 ms to 2,800 ms. Participants were instructed to respond as quickly and accurately as possible to the direction of the middle arrow. If the middle arrow was pointing to the right, participants were told to click the right mouse button. Similarly, if the middle arrow was pointing to the left, participants were instructed to click the left mouse button. Half of the trials involved congruent flankers (<<<<<, >>>>>) and the other half of trials involved incongruent flankers (<<><<, >><>>). The order of these trials was random. All participants completed a 10-trial practice block before starting the actual task to assure competence. More specifically, participants had to respond correctly to at least 70% of the practice trials to continue to the actual task. The full task consisted of a total of 330 trials (i.e., 11 blocks of 30 trials). Participants received feedback after each block based on their recent performance in the block. If the accuracy was low (i.e., 75% or below), they would receive the message, “Please try to be more accurate.” High accuracy scores (i.e., 90% or above), resulted in the message, “Please try to respond faster.” Accuracy between these thresholds (i.e., 75% to 90%), received the message, “You’re doing a great job.”

#### EEG recording and processing

EEG was recorded employing a 32-channel active ActiCap slim electrode (Ag/AgCl) actiCHamp system (Brain Products GmbH, Gilching, Germany) at each assessment. The 32-electrode array was in accordance with the international 10/20 electrode system. Electrode site Cz served as the online recording reference and additional electrodes were placed on the left and right mastoids to serve as offline references. Four additional electrodes were utilized for EOG: one placed above and below the left eye, and one to the sides of each eye. EEG data were digitized at a sampling rate of 1000 Hz and the low-pass online filter was set at 100 Hz.

EEG analyses were performed offline using Brain Vision Analyzer (version 2.2; Brain Products, Gilching, Germany). EEG data were first re-referenced to average mastoids. Next, the EEG data were filtered with low and high filter cutoffs set at 0.01 Hz (4^th^ order) and 30 Hz (4^th^ order), respectively. Next, ocular artifacts were corrected employing the Gratton & Coles (1983) procedure. Next, automatic artifact rejection was implemented of EEG data which rejected any data with a voltage step greater than 50 µV, a voltage difference of 175 µV within a 600 ms interval, or a maximum voltage difference of less than 0.50 µV within 100 ms intervals. On average, participants in the current sample retained the vast majority of trials even after artifact rejection. More specifically, 0.45% of trials (*SD* = 2.70) were excluded at baseline (T1), 1.61% (*SD* = 10.55) of trials were excluded at T2, and 0.88% (*SD* = 7.45) were excluded at T3. Furthermore, percentage of rejected trials was not significantly associated with depressive or anxiety symptoms at any assessment, *r*’s < 0.09, *p*’s > .06.

#### P300 quantification

**Figure 1.**
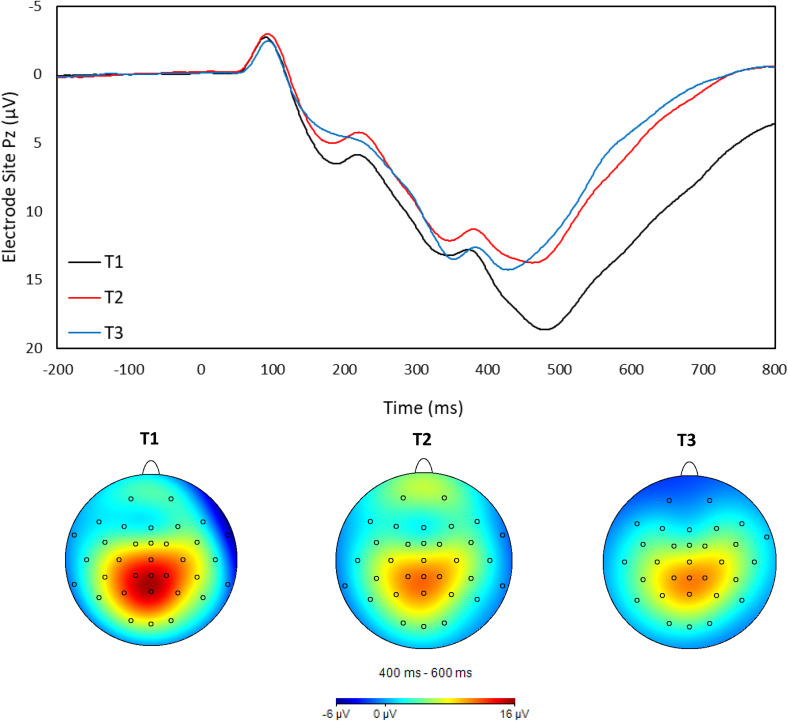
Flanker stimulus-locked grand average waveforms and head maps of the sample at the baseline (T1), two-month (T2), and two-year (T3) assessments.

EEG data was then segmented starting 200 ms before the stimulus presentation to 800 ms after presentation on trials in which participants responded correctly to the direction of the middle arrow. Segments were then baseline-corrected using the 200 ms pre-stimulus interval and averaged across all correct congruent and incongruent trials. P300 amplitude was quantified as the mean amplitude occurring 400 to 600 ms after presentation of the flanker stimuli at electrode site Pz (see Santopetro et al., 2020, 2021). Figure 1 depicts grand averaged flanker stimuli-locked neural activity and topographic head maps in the entire sample at each assessment.

The correlations between P300 amplitude on odd and even trials were examined at each assessment to measure internal consistency (Levinson et al., 2017) and corrected using the Spearman-Brown prophecy formula (Nunnally et al., 1967). The P300 exhibited excellent internal consistency at each assessment (*T1*: .98; *T2*: .98; *T3*: .98). Additionally, P300 amplitude demonstrated good test-retest reliability over two months (*r* = .73; T1 to T2) and two years (*r* = .69; T1 to T3).

### Statistical analyses

All data analyses were performed in *R* version 4.0.5 (*R* Core Team, 2021) and *jamovi* version 2.3 (The jamovi project, 2022) with a significance criterion of *α* = .05 (two-tailed). In particular, the “lme4” (Bates, Mächler, Bolker, & Walker, 2014) and “lmerTest” (Kuznetsova et al., 2017) *R* packages were used to fit the MLMs. We first conducted bivariate correlations (Pearson’s *r*) to examine associations between flanker P300 amplitude at each assessment and self-reported internalizing symptoms at each assessment (i.e., CDI total score and SCARED total score). Next, we conducted a multilevel model (MLM) to simultaneously evaluate whether between-subjects (i.e., more trait-like) or within-subjects (i.e., more state-like) symptoms of depression and/or anxiety were linked to flanker P300 amplitude at each timepoint after covarying for participant age, sex, and AABM training group. At level 1 (repeated observations), depressive symptoms (person-mean centered), anxiety symptoms (person-mean centered), and age at each assessment were employed as predictors. At level 2 (each participant), models included average depressive symptoms over the two-year period (grand-mean centered), average anxiety symptoms over the two-year period (grand-mean centered), sex (0 = male, 1 = female), and AABM training status (0 = no training, 1 = training). The first model was conducted *without* the inclusion of important covariates (i.e., age, sex, and AABM training) but was then followed by an additional sensitivity analysis including the covariates. An additional sensitivity analysis was also conducted employing only individuals with data at all three timepoints. To determine the most appropriate and parsimonious model specification of fixed and random effects, we first estimated an unconditional model in which the intercept was allowed to vary across participants (i.e., random intercept) with fixed slopes. We then estimated two additional models that included random slopes for within-subject depressive and anxiety scores, respectively. Likelihood ratio tests indicated that models including both a random intercept and random slope did not provide a better fit compared to the models with random intercepts and fixed slopes. Indeed, this empirical approach is in line with past research that utilizes likelihood ratio tests to determine the most appropriate and parsimonious multilevel models (Volpert-Esmond et al., 2021). As a result, all final models included a random intercept, with all other effects modeled as fixed. For all models, Satterthwaite approximations were used to estimate degrees of freedom and to obtain two-tailed *p*-values, with maximum likelihood used to estimate model parameters.

## Results

### Zero-order correlations and *t*-tests

Average depressive and anxiety symptom scores over the two-year period were strongly associated with each other, *r*(488) = .76, *p* < .001. Participants with reduced P300 amplitudes on average reported more depressive symptoms on average, *r*(488) = −.24, *p* < .001, and anxiety symptoms on average, *r*(488) = −.18, *p* < .001.^1^ Older adolescents indicated more symptoms of depression on average, *r*(488) = .24, *p* < .001, more symptoms of anxiety on average, *r*(488) = .20, *p* < .001, and smaller P300 amplitude on average, *r*(488) = −.32, *p* < .001. Female adolescents reported significantly more symptoms of depression, *t*(488) = 5.74, *p* < .001, and anxiety, *t*(488) = 8.24, *p* < .001, on average compared to males. Females displayed smaller P300 amplitude compared to males at baseline (T1), *t*(488) = −2.37, *p* = .018; there were no significant sex differences in flanker P300 amplitude at the T2, *t*(444) = 0.52, *p* = .603, or the T3 assessments, *t*(193) = 1.56, *p* = .120. Table 2 provides more information regarding zero-order correlations between P300 amplitude, CDI total score, SCARED total score, and age at each assessment.

**Table 2.** Flanker P300 and age correlations with internalizing symptoms

Variable	1. T1 CDI	2. T2 CDI	3. T3 CDI	4. T1 SCARED	5. T2 SCARED	6. T3 SCARED
1. T1 P300 (µV)	−.23***	−.25***	−.20***	−.17***	−.17**	−.19***
2. T2 P300 (µV)	−.17***	−.18***	−.12*	−.11*	−.12*	−.09
3. T3 P300 (µV)	−.16*	−.21**	−.17*	−.12	−.11	−.15*
4. T1 Age (Years)	.22***	.20***	.23***	.17**	.17**	.22***
5. T2 Age (Years)	.22***	.20***	.24***	.18***	.17***	.22***
6. T3 Age (Years)	.22***	.21***	.23***	.19***	.18**	.23***

*Note.* CDI represents the Child’s Depression Inventory total score. SCARED represents the Screen for Child Anxiety Related Emotional Disorders total score. * indicates *p* < .05. ** indicates *p* < .01. *** indicates *p* < .001.

### MLMs

There was no significant difference in the overall model fit when utilizing a RI-FS model as compared to a RI-RS model (utilizing within-subject depressive symptoms for slope), Δ*X*^*2*^ = 0.037, *p* = .981, and no significant difference in model fit between RI-FS model and RI-RS model (utilizing within-subject anxiety symptoms for slope), Δ*X*^*2*^ = 0.038, *p* = .981. There was also no significant difference in model fit between random intercept, random slope (RI-RS) model (utilizing within-subject depressive symptoms for slope) and the RI-RS model (utilizing within-subject anxiety symptoms for slope), Δ*X*^*2*^ = 0.00, *p* = .999. Therefore, results suggest that a random intercept, fixed slope (RI-FS) model best fits the current data.

For the unconditional model, the mean intercept (*b* = 12.40, SE = 0.26, *t*[491] = 47.50, *p* < .001) was significant. The coefficient ICC of the model was 0.577, suggesting that approximately 57.7% of the overall variance in P300 amplitude could be attributed to between-subjects variability, while the remaining 42.3% was accounted for by within-subjects variability. The conditional model predicting P300 amplitude was specified as:

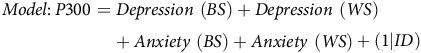

*Note.* “BS” represents between-subject and “WS” represents within-subject.

In this conditional model, we simultaneously examined associations between within-subject and between-subject depressive and anxiety symptoms with P300 amplitude. The full model is reported in detail in Table 3. There was a significant between-subject effect of depressive symptoms, indicating that adolescents reporting more depressive symptoms exhibited smaller P300 amplitudes, on average, while there was no within-subject effect. Conversely, there was an opposing pattern for anxiety symptoms, such that there was a within-subject effect on P300 amplitude, indicating that experiencing more anxiety symptoms at a particular study visit was related to a larger P300 amplitude at that study visit. There was no between-subject effect of anxiety with P300 amplitude.^2^ We conducted a sensitivity analysis including only participants with data at all three assessments. Overall, the results showed similar patterns as the original model (see Table 4).

**Table 3.** Multilevel model predicting P300 amplitude utilizing between- and within-subject internalizing symptoms in participants with data at two or more assessments (*n* = 490)

*Fixed effects*	*b*	*SE*	*95%* CI	*df*	*t*	*p*
Intercept	12.38	0.26	[11.88, 12.88]	482	48.48	<.001
**Depression BS**	**−0.21**	**0.06**	**[−0.34, −0.09]**	**495**	**−3.32**	**<.001**
Depression WS	−0.01	0.05	[−0.11, 0.09]	698	−0.16	.877
Anxiety BS	−0.00	0.03	[−0.07. 0.06]	488	−0.10	.917
**Anxiety WS**	**0.07**	**0.03**	**[0.02, 0.12]**	**680**	**2.55**	**.011**

*Note.* Depression BS = mean-centered CDI total score averaged across the three timepoints (between-subject). Depression WS = person-centered CDI total scores (within-subject). Anxiety BS = mean-centered SCARED total score averaged across the three timepoints (between-subject). Anxiety WS = person-centered SCARED total scores (within-subject). Bold items indicate a significant *p*-value that is less than .05.

**Table 4.** Multilevel model predicting P300 amplitude utilizing between- and within-subject internalizing symptoms in participants with data at all three assessments (*n* = 146)

*Fixed effects*	*b*	*SE*	*95%* CI	*df*	*t*	*p*
Intercept	12.42	0.47	[11.48, 13.36]	146	26.62	<.001
**Depression BS**	**−0.30**	**0.14**	**[−0.54, −0.02]**	**146**	**−2.14**	**.034**
Depression WS	−0.10	0.08	[−0.26, 0.04]	292	−1.37	.171
Anxiety BS	0.05	0.07	[−0.09. 0.19]	146	0.80	.426
**Anxiety WS**	**0.08**	**0.04**	**[0.16, 0.00]**	**292**	**1.96**	**.050**

*Note.* Depression BS = mean-centered CDI total score averaged across the three timepoints (between-subject). Depression WS = person-centered CDI total scores (within-subject). Anxiety BS = mean-centered SCARED total score averaged across the three timepoints (between-subject). Anxiety WS = person-centered SCARED total scores (within-subject). Bold items indicate a significant *p*-value that is less than .05.

Lastly, we conducted an additional sensitivity analysis to examine whether the above effects explained P300 amplitude over and above age, sex, and AABM training status, which were included as covariates. The results followed a similar and consistent pattern as the models without covariates. In this model, the between-subject effect of depressive symptoms with P300 amplitude was significant, as was the within-subject effect of anxiety symptoms with P300 amplitude. Participant age was also a significant predictor of P300, such that older adolescents exhibited smaller P300 amplitude. Lastly, sex and AABM training group status did not significantly relate to P300 amplitude in the model (Table 5).^3^

**Table 5.** Multilevel model predicting P300 amplitude utilizing between- and within-subject internalizing symptoms controlling for age, sex, and AABM training (*n* = 490)

*Fixed effects*	*B*	*SE*	*95%* CI	*df*	*t*	*p*
Intercept	34.55	1.91	[30.73, 39.37]	1106	18.11	<.001
**Depression BS**	**−0.14**	**0.06**	**[−0.27, −0.02]**	**503**	**−2.34**	**.020**
Depression WS	0.04	0.05	[−0.06, 0.14]	691	0.80	.425
Anxiety BS	0.01	0.03	[−0.06. 0.07]	494	0.32	.751
**Anxiety WS**	**0.07**	**0.02**	**[0.02, 0.12]**	**671**	**2.80**	**.005**
**Age WS**	**−1.65**	**0.14**	**[−1.92, −1.38]**	**1103**	**−12.01**	**<.001**
Sex	−0.45	0.53	[−1.51, 0.61]	487	−0.85	.394
Training group	0.15	0.51	[−0.86, 1.14]	486	0.30	.768

*Note.* Depression BS = mean-centered CDI total score averaged across the three timepoints (between-subject). Depression WS = person-centered CDI total scores (within-subject). Anxiety BS = mean-centered SCARED total score averaged across the three timepoints (between-subject). Anxiety WS = person-centered. SCARED total scores (within-subject). Age WS is within-subject. Sex is coded 0 = male, 1 = female. Training Group is coded 0 = no AABM training, 1 = AABM training. Bold items indicate a significant *p*-value that is less than .05.

## Discussion

In the current study we examined relationships between the P300 with both depressive and anxiety symptoms by investigating between- and within-subject effects across three visits over a two-year period. We found unique effects, such that between-subject depressive symptoms significantly related to *smaller* P300 amplitude, while between-subject anxiety symptoms did not. However, within-subject increases in anxiety relative to that individual’s average anxiety symptoms were associated with *a larger* P300 amplitude for that time point while within-subject fluctuations in depressive symptoms were not associated with the P300. These findings remained consistent even when controlling for important factors such as participant age, sex, AABM training status, or data collection site.

The current P300 and depression findings are in line with more historic findings employing oddball tasks suggesting that adults suffering from depressive disorders are characterized by smaller P300 amplitude (see Bruder et al., 2012; Santopetro et al., 2025 for review). Additionally, these findings are in line with more recent studies that have continued to explore the P300 and depression relationship utilizing other experimental paradigms such as the flanker task. Indeed, recent work observed that flanker stimulus-locked P300 amplitude was reduced in adults experiencing a current depressive disorder compared to controls with no history of depression (Klawohn et al., 2020). Moreover, current findings are consistent with more limited P300 findings in youth samples that find that blunted flanker P300 amplitude is related to both current depressive symptoms (Santopetro et al., 2021) as well as risk for further increases in depressive symptoms over two years in adolescents (Santopetro et al., 2020). Importantly, the current project leverages longitudinal data to simultaneously examine between- and within-subject variance related to depression and anxiety but does not attempt to understand changes or trajectory of these symptoms over time.

Despite this evidence that P300 amplitude deficits are linked to depression in both adults and youth, it remained largely unclear if P300 impairments are reflecting between-subject levels of symptoms or within-subject fluctuations in depressive symptoms. The current results indicate that P300 deficits observed in youth depression are more robustly associated with between-subject symptoms of depression and that this effect does not vary according to changes in the individual’s state symptoms of depression (i.e., within-subject level). Therefore, the P300 component is likely indexing stable tendency or risk to experience depressive symptomology rather than current fluctuations or increases in an individuals’ symptoms. Considering that depression is an extremely heterogeneous disorder with varying presentations (Zimmerman et al., 2015), identifying neural correlates linked to more pervasive and stable patterns of depression could assist with early identification and treatment efforts. It will be critical for future investigations to continue to replicate these findings utilizing advanced statistical methods in combination with longitudinal designs consisting of multiple assessments. For example, another recent study employing MLM analyses similarly observed that reductions in P300 amplitude elicited from the flanker task were particularly associated with between-subject, not within-subject, elevations in anhedonia symptoms in a sample of young adult females that completed four assessments over the course of one month (Santopetro et al., 2022).

Present findings regarding anxiety symptoms and P300 amplitude in youths add further insights into this overall mixed literature. Specifically, our findings are somewhat in line with past adult and youth investigations that suggest anxiety symptomology is characterized by *larger* P300 amplitude (Bruder et al., 2002; Enoch et al., 2001, 2008; Singhal et al., 2012). In particular, previous research has observed associations between the P300 and anxiety-related hyperactivity of PFC, suggesting increased cognitive resource allocation to task-relevant stimuli in anxiety (Li et al., 2015). However, it is important to note that previous investigation have primarily focused on *between-subject* relationships regarding P300 amplitude and anxiety, while the current findings indicate that the P300 is elevated on occasions wherein youth experience increased anxiety relative to their typical levels (i.e., within-subject effect). Therefore, an elevated P300 amplitude might reflect a transient physiological reaction (i.e., state anxiety) rather than serve as a marker of individual differences associated with risk to present with state anxiety (i.e., trait anxiety; Leal et al., 2017). Indeed, these findings are consistent with recent research that specifically examined state anxiety associations with the P300 suggesting that elevations in P300 amplitude characterize higher state anxiety (Ioakeimidis et al., 2021; Rowe et al., 2021). It is important to highlight that these studies quantified state anxiety by utilizing self-report measures such as the State-Trait Anxiety Inventory (STAI) and were conducted in adult samples. In sum, results suggest that elevated attention allocation to imperative stimuli (i.e., greater P300 amplitude) is better attributed to more state-like symptoms of anxiety (i.e., hyperarousal) rather than serving as a trait-like neural correlate of anxiety as some past studies have suggested.

Examination of co-occurring anxiety symptoms in relation to the depression and P300 amplitude relationship adds clarity to the tripartite model of depression and anxiety. Although we found that anxiety symptoms relate to a reduced P300 amplitude in zero-order correlations, which stands in contrast to past adult and youth investigations that suggest anxiety symptoms relate to a *larger* P300 amplitude (Bruder et al., 2002; Enoch et al., 2001, 2008; Singhal et al., 2012), we found that accounting for depression in the same model indicates that the unique variance explained by anxiety symptoms regarding P300 amplitude may be due to occasion-specific effects (i.e., *within-subject*), rather than individual differences in anxiety symptoms (i.e., between-subject). After accounting for both between- and within-subject effects of depression and anxiety symptoms on P300 amplitude, we found that within-subject fluctuations in anxiety symptoms related to larger P300 amplitude, which is more consistent with research showing that higher state anxiety is associated with larger P300 amplitude (Ioakeimidis et al., 2021; Rowe et al., 2021). The current study highlights the importance of examining and controlling for additional clinical characteristics. Initial zero-order results in the present sample suggested that elevations in anxiety symptoms were associated with *smaller* P300; however, it is possible that this initial relationship may be better explained by co-occurring depressive symptoms that are highly comorbid with anxiety (i.e., depressive and anxiety symptoms were moderately correlated in the current sample at each assessment; *r’s* ranging from .44 to .74, *p’s* < .001). It is also plausible that shared variance between depression and anxiety, elevated negative affectivity, may be contributing to reduced P300 amplitude. It is important to highlight that multicollinearity was not a significant issue in the current sample (see footnote 2). Therefore, inconsistencies in some past P300 and anxiety findings could stem from not properly examining and/or controlling for overlapping symptoms of current depression.

Youth depressive and anxiety symptoms related to P300 amplitude in unique and divergent patterns. As reviewed above, between-subject symptoms of depression were associated with *smaller* P300 amplitude while within-subject symptoms of anxiety reflected *larger* P300 amplitude. The distinct functionality of the P300 in youth depression and anxiety appear to be in line with past conceptualizations which outlines common aspects shared by these internalizing issues as well as identifies aspects that uniquely characterize each disorder from one another. Indeed, some studies suggests that deficits in P300 amplitude observed in depression might specifically reflect anhedonia, loss of interest or pleasure in activities (i.e., low positive affect), and that depression-related reductions in the P300 potential are in part attributed to motivational deficits to engage with the experimental paradigm (Ancy et al., 1996; Gangadhar et al., 1993; Kallen et al., 2025; Santopetro et al., 2020, 2021b, 2022; Urretavizcaya et al., 2003). Furthermore, findings from samples suffering from particular anxiety-related disorders such as PTSD observe specific associations between elevated symptoms of hyperarousal and larger P300 amplitude (Bae et al., 2011; Kimura et al., 2013). However, it is also critical to highlight that reduced P300 amplitude may be attributed to shared negative affectivity between depression and anxiety. Indeed, additional research suggests that blunted P300 might more broadly characterize higher order internalizing issues as well as a general factor of psychopathology (Bernat et al., 2020; Pasion et al., 2023; Trayvick et al., 2024). Taken together, the current findings indicate that the P300 ERP component can potentially serve as both a marker of trait-like deficits in positive affect as well as a marker of fluctuation in state-like hyperarousal.

The psychometric properties of the P300 ERP elicited from the oddball and flanker tasks have been examined in previous publications in adult samples with results suggesting good between- and within-session reliability (Fabiani et al., 1987; Santopetro et al., 2022; Segalowitz & Barnes, 1993; Sklare & Lynn, 1984). Findings from the current study extend this important psychometric work to the flanker P300 component measured during adolescence. A major concern in neuroscience research is that neural measures with poor psychometric properties can either over- or underestimate associations with other variables and are therefore not well-suited to be leveraged as measures to better understand mental health disorders (Clayson et al., 2021). The flanker P300 in the current sample demonstrated excellent internal consistency at all three assessments (.98) and good test-retest reliability over both two months (.73) and two years (.69). The psychometric properties of the P300 component were on par with, if not superior to in some instances, the well-established self-report questionnaires employed to measure depressive (i.e., CDI) and anxiety symptoms (i.e., SCARED) in the current sample. In sum, the flanker P300 when measured during adolescence exhibits strong psychometric properties, evidenced by its internal consistency and test-retest reliability, which provides further support for its utility as an individual difference measure in future investigations.

The present study has limitations worth discussing. There was limited ethnic, racial, and socioeconomic diversity in the current sample limiting generalizability of the current findings. Lack of representation (i.e., ethnic, racial, gender, SES) in EEG/ERP research is an important problem that greatly hinders establishing meaningful normative data (Choy et al., 2022). Furthermore, the majority of adolescents in the current sample were relatively “healthy” in terms of psychopathology at each assessment, reporting low symptoms of depression and anxiety below clinical thresholds according to the CDI and SCARED, respectively. Lastly, the current project did not investigate potential between- and within-subject effects of externalizing symptoms on P300 amplitude primarily due to logistical limitations (i.e., externalizing measures were collected in only half of the sample at just two timepoints). Indeed, externalizing psychopathology has been consistently linked to smaller P300 (Iacono et al., 2003; Patrick et al., 2006; Perkins et al., 2024) and it remains uncertain if these P300 dysfunctions are discrete from depression-related deficits. It is therefore imperative for future studies to investigate the relationships between flanker P300 amplitude, depression, anxiety, and externalizing psychopathology in youth samples experiencing clinical levels of these symptoms.

In sum, the current study is among the first to employ advanced statistical methods (MLM) to disaggregate between- and within-subject effects of internalizing symptoms with P300 during adolescence. Findings from these statistical models suggest distinct and differing relationships of between- and within-subject internalizing symptoms while simultaneously employed as predictors of the P300 ERP component. More specifically, elevations in between-subject symptoms of depression were associated with *blunted* P300 amplitude while higher within-subject anxiety symptoms related to *larger* P300 amplitude; neither within-subject variance in depressive symptoms nor between-subject variance in anxiety symptoms significantly related to P300 amplitude. These differing neural associations are directly in line with low positive affect specific to depression and hyperarousal specific to anxiety which have been theorized to differentiate these internalizing problems. These findings add to the growing body of literature on the P300 in youth internalizing disorders and suggest that P300 might be a valuable neural measure that can continue to be leveraged to further elucidate the neurophysiological underpinnings which differentiate depressive and anxiety disorders.

## Data Availability

All data from the present project have been posted to the NIMH Data Archive (NDA) and will be available after embargo. Alternatively, materials can be shared upon request with the first author.

## Footnotes

1 Flanker congruency did not affect the relationship between average P300 amplitude and average depressive or anxiety symptoms: elevated depressive symptoms on average were significantly associated with reduced average P300 amplitude on both congruent trials, *r*(488) = −.24, *p* < .001, and incongruent trials, *r*(488) = −.22, *p* < .001, and increased anxiety symptoms on average were significantly associated with a reduced average P300 amplitude on congruent, *r*(488) = −.19, *p* < .001, and incongruent trials *r*(488) = −.17, *p* < .001.

2 To examine multicollinearity, we conducted three multiple linear regressions predicting T1 P300, T2 P300, and T3 P300 amplitude employing average depressive and anxiety symptoms over the three timepoints as predictors to examine variance inflation factor (VIF) and tolerance. Results from the three models indicated acceptable VIF scores (2.25–3.08) and tolerance scores (0.33–0.45) suggesting that multicollinearity was not a significant issue when employing depressive and anxiety symptoms as independent variables in the same models.

3 Data collection site (0 = FSU, 1 = SDSU) was included as an additional fixed binary covariate into the model. The results followed a similar and consistent pattern as the models without covariates and the original sensitivity analysis. In this model, the between-subject effect of depressive symptoms with P300 amplitude was significant, as was the within-subject effect of anxiety symptoms with P300 amplitude. Participant age was also still a significant predictor of P300, such that older adolescents demonstrated smaller P300 amplitude. Lastly, sex, AABM training group status, and data collection site did not significantly relate to P300 amplitude in the model.

## Pre-registration statement

The present study was not preregistered as it involved secondary data analysis of a known dataset.
